# Comparing the performance of air pollution models for nitrogen dioxide and ozone in the context of a multilevel epidemiological analysis

**DOI:** 10.1097/EE9.0000000000000093

**Published:** 2020-05-13

**Authors:** Barbara K. Butland, Evangelia Samoli, Richard W. Atkinson, Benjamin Barratt, Sean D. Beevers, Nutthida Kitwiroon, Konstantina Dimakopoulou, Sophia Rodopoulou, Joel D. Schwartz, Klea Katsouyanni

**Affiliations:** a Population Health Research Institute, St George’s, University of London, London, United Kingdom; b Department of Hygiene, Epidemiology and Medical Statistics, Medical School, National and Kapodistrian University of Athens, Athens, Greece; c MRC Centre for Environment and Health, King’s College London, London, United Kingdom; d National Institute for Health Research Health Protection Research Unit (NIHR HPRU) in Health Impact of Environmental Hazards, King’s College London, London, United Kingdom; e School of Population Health and Environmental Sciences and MRC Centre for Environment and Health, King’s College London, London, United Kingdom; f Department of Environmental Health, Harvard School of Public Health, Boston, Massachusetts, USA.

## Abstract

Using modeled air pollutant predictions as exposure variables in epidemiological analyses can produce bias in health effect estimation. We used statistical simulation to estimate these biases and compare different air pollution models for London.

**Methods:**

Our simulations were based on a sample of 1,000 small geographical areas within London, United Kingdom. “True” pollutant data (daily mean nitrogen dioxide [NO_2_] and ozone [O_3_]) were simulated to include spatio-temporal variation and spatial covariance. All-cause mortality and cardiovascular hospital admissions were simulated from “true” pollution data using prespecified effect parameters for short and long-term exposure within a multilevel Poisson model. We compared: land use regression (LUR) models, dispersion models, LUR models including dispersion output as a spline (hybrid1), and generalized additive models combining splines in LUR and dispersion outputs (hybrid2). Validation datasets (model versus fixed-site monitor) were used to define simulation scenarios.

**Results:**

For the LUR models, bias estimates ranged from −56% to +7% for short-term exposure and −98% to −68% for long-term exposure and for the dispersion models from −33% to −15% and −52% to +0.5%, respectively. Hybrid1 provided little if any additional benefit, but hybrid2 appeared optimal in terms of bias estimates for short-term (−17% to +11%) and long-term (−28% to +11%) exposure and in preserving coverage probability and statistical power.

**Conclusions:**

Although exposure error can produce substantial negative bias (i.e., towards the null), combining outputs from different air pollution modeling approaches may reduce bias in health effect estimation leading to improved impact evaluation of abatement policies.

## Abstract 2

Supplemental Digital Content is available in the text.

**What this study adds**

This study demonstrates how statistical simulation methodology can be employed to compare the performance of different air pollution models in terms of their use in providing exposure variables for complex epidemiological analyses of air pollution and health. It illustrates that combining outputs from different models, such as those based on land use regression or dispersion, maybe a way forward in reducing bias in health effect estimation and preserving coverage probability and statistical power. It also highlights the potential benefits of combining such outputs using generalized additive models (GAM).

## INTRODUCTION

Exposure estimates from spatio-temporal air pollution models are commonly used as exposure variables in epidemiological analyses of air pollution and health. However, measurement error may be introduced into model predictions due to oversmoothing the pollutant surface (i.e., Berkson-like error), and classical-like error may be introduced due to model parameter prediction.^1^ The magnitude of these errors is generally assessed using data from validation studies comparing monitor and model outputs and calculating standard metrics such as the residual mean square error.^2–4^ These metrics are informative about the level of bias in individual exposure estimates, but less informative when trying to assess the total adverse impact of measurement error on effect estimation in epidemiological analyses of air pollution and health.

This has led to the use of statistical simulation as an alternative approach to assessing pollution model performance.^1,5–9^ Although some of these studies have observed marked negative bias (i.e., towards the null) in health effect estimation due to additive classical error in model outputs,^5–9^ others have observed some positive bias (i.e., away from the null) if the Berkson component of error is additive on a log scale.^5–7^ However, a simulation study by Szpiro et al,^1^ investigating the use of exposure predictions from a land use regression (LUR) model in a linear regression analysis, observed little difference in health effect bias when the accuracy of exposure predictions was compromised by dropping an important geographic variable from the LUR. Although this suggests that improving the accuracy of exposure prediction may not improve health effect estimation, whether we would observe similar results under newly proposed approaches to pollution modeling or more complex outcome analyses is unclear and merits investigation.

As part of the project entitled, “Comparative evaluation of Spatio-Temporal Exposure Assessment Methods for estimating health effects of air pollution” (STEAM), we use statistical simulation methods, described in our previous article,^5^ to assess the impact of measurement error introduced by using model outputs as exposures in a single pollutant multilevel epidemiological analysis. Our aim is to compare the performance of different London based pollutant models for NO_2_ and O_3_. The models were developed using 4 different modeling approaches, namely LUR, dispersion, and two hybrid models combining both techniques (hybrid1 and hybrid2).

## Methods

The context of our simulations is a sample of 1,000 Lower Super Output Areas (LSOA) within the London M25 orbital motorway and a spatio-temporal epidemiological analysis conducted at the LSOA level, over the period 2009–2013, and facilitating the joint estimation of health effects from both short-term (daily mean) and long-term (5-year mean) pollutant exposures.^10^ An LSOA is a small geographic area, with an average population of approximately 1,500 residents.^11^ Our simulations consider scenarios each defined by a combination of outcome measure (all-cause mortality or cardiovascular hospital admissions), pollutant (NO_2_, O_3_), error type (additional, proportional), pollution model (LUR, dispersion, hybrid1, and hybrid2), and site type (urban/suburban background or roadside/kerbside). The inclusion of two outcome measures allowed us to investigate the effect of changing the baseline disease rate and the underlying concentration-response functions.

### Monitor data

Daily measurements of the gaseous pollutants were obtained for 2009–2013 from NO_2_ monitoring sites within the M25 London road network and O_3_ monitoring sites within the wider southeast region. NO_2_, data were available from 72 roadside/kerbside sites and 47 urban/suburban background sites. For O_3_, the corresponding figures were 10 and 36. These data were obtained from the London Air Quality Network,^12^ and Air Quality England,^13^ and include data from the Automatic Urban and Rural Network (AURN).^14^

#### Meteorological data

Meteorological related variables used to inform pollutant models were obtained from the UK Met Office through the Centre for Environmental Data Analysis (CEDA).^15^

### Pollutant modeling

#### Land use regression

We developed spatio-temporal semiparametric models, of the form:


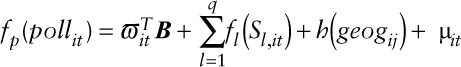


where 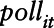 is the measurement of the air pollutant at location 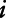 on day 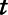, 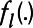 is an unspecified smooth function reflecting the nonlinear effect of covariate 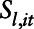 on the transformed pollutant concentration 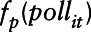, 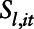 stands for the 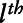 smoothed covariate; 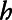 is a bivariate smooth function of geographical coordinates (latitude and longitude) accounting for residual correlation between locations 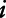 and 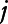; **ϖ**_it_ is the vector of covariates that have a linear effect on 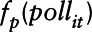; ***B*** is the corresponding vector of regression coefficients; and 
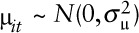
. For NO_2_, 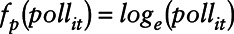 and for O_3_, 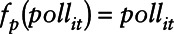


#### Dispersion

The Community Multiscale Air Quality (CMAQ-urban) model^16,17^ combines emissions data with the Weather Research and Forecasting (WRF) meteorological model v3.6.1 (National Centre for Atmospheric Research, Boulder, CO),^18^ and the Community Multiscale Air Quality (CMAQ) model v5.0.2 (U.S. Environmental Protection Agency, Washington, DC),^19^ which has been coupled to the Atmospheric Dispersion Modeling System (ADMS) roads model v4 (Cambridge Environmental Research Consultants, Cambridge, UK).^20^ For this study, the anthropogenic emissions data were obtained by combining the UK National Atmospheric Emissions Inventory (NAEI),^21^ the London Atmospheric Emissions Inventory,^22^ King’s road transport emissions model,^23^ and the European Monitoring and Evaluation Programme European emissions.^24^ The biogenic emissions from vegetation and soils were estimated using the Biogenic Emission Inventory System version 3 (BEIS3) model (U.S. Environmental Protection Agency).^25^ Sea-salt emissions were calculated in line in CMAQ. The CMAQ-urban model outputs hourly air pollution concentrations at 20 m grid resolution across study domain. The model provides nitrogen oxides (NO_X_), NO_2_ and O_3,_ with the ADMS roads model used to describe the near field dispersion from roadways and NO_2_ and O_3_, using a simple chemical scheme.^26^

#### Hybrid models

Hybrid1: For each pollutant, we constructed a combined LUR-dispersion model by incorporating into the LUR, daily predicted air pollutant values estimated from the CMAQ-urban dispersion model at fixed-site air pollution monitoring locations, as a nonlinear covariate. The resulting models took the form:


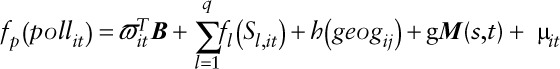


where 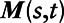 is a spatio-temporal spline representing the CTM model predictions with coefficient, 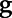.

Hybrid2: For each pollutant, using R version 3.3.3 (R Foundation for Statistical Computing, Vienna, Austria),^27^ and library mgcv with generalized cross-validation smoothing,^28^ a generalized additive model (GAM) approach was applied to combine predicted pollutant concentrations at fixed-site monitoring locations from the developed spatio-temporal LUR and CMAQ-urban dispersion models. The GAM was developed by fitting two corresponding splines of the predicted variables to measurements at fixed monitoring sites. For the LUR, we used 10-fold cross-validated predictions.

### Validation data

For dispersion modeling, validation data consisted of model NO_2_ and O_3_ predictions for 2009–2013 for all monitoring sites, linked to their corresponding monitor measurements, which played no role in the modeling. For models that included monitoring data in the modeling process (i.e., LUR, hybrid1, and hybrid2), a 10% leave-out rule was used by which 10% of monitors were omitted, the model recalibrated, and used to predict pollutant outputs at the left-out sites. This process was repeated until a full model-monitor dataset was achieved, predicting values for the complete set of monitors.

### Simulation strategy

Following the same general approach as detailed in our previous article,^5^ our simulation strategy consisted of 4 basic steps:

Step1: Simulating “true” daily mean outdoor air pollutant data for the geographic centroid of each LSOA using a simple pollutant site-type specific spatio-temporal model developed from monitor measurements in our validation datasets. As in our previous article,^5^ the model incorporated spatio-temporal variances and covariances as well as adjusting for instrument error in the monitor measurements.

Step2: Simulating “true” outcome data from the “true” pollutant data, incorporating a relationship between the two based on a multilevel Poisson regression model,^10^ with three prespecified regression coefficients representing: baseline disease rate 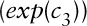; the short-term concentration-response function (CRF) per 1 µg/m^3^ change in pollutant (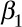); and the long-term CRF per 1 µg/m^3^ change in pollutant (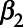). The values of these coefficients used for each pollutant and outcome combination are listed in eTable 1; http://links.lww.com/EE/A86.

Step3: Simulating pseudo-modeled daily pollutant data from the “true” pollutant data prespecifying both the temporal (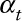) and spatial (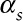) Pearson correlation coefficients between the two and their temporal (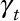) and spatial (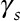) variance ratios (model versus “true”). For each pollution model, pollutant, and site type, these parameters 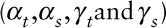 were estimated from an analysis of validation data with correction for instrument error in monitor measurements as described in ePage 3; http://links.lww.com/EE/A86.

Step4: Refitting the multilevel Poisson regression model, replacing “true” pollutant data with the corresponding pseudo-modeled data. This provides us with estimates of 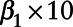 and 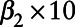 (i.e., expressed per 10 µg/m^3^) and their corresponding standard errors.

For NO_2_, we considered not only additive measurement error but proportional error (i.e., additive on a log scale). Proportional error scenarios use log_e_(NO_2_) as the pollutant leading to the simulation of “true” log_e_(NO_2_) data and pseudo-modeled log_e_(NO_2_) data. Simulated “true” and pseudo-modeled log_e_(NO_2_) data are back-transformed to NO_2_ for simulating “true” outcome data and refitting the Poisson regression model, respectively.

Simulations were run in R version 3.4.3,^27^ using libraries Hmisc,^29^ lme4,^30^ MASS,^31^ and foreign.^32^

#### Performance assessment

For each combination of pollutant (NO_2_ [with additive or proportional error], O_3_ [with additive error]), site type, pollution model, and outcome, we ran 1,000 simulations and obtained 1,000 estimates of: 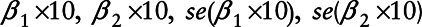, from which we calculated, for both long and short-term exposure, the average health effect estimate, average standard error, percent bias in health effect estimation, coverage probability as the percentage of 95% confidence intervals containing the true concentration-response function, and power as the percentage of significance tests that were statistically significant at the 5% level.^33^ Using our simulated health effect estimates, we tested for differences from their respective “true” values by calculating simple one-sample t-tests.

### Standard performance metrics

For each pollutant, pollution model, and site type, we also calculated: mean bias; normalized mean bias; normalized mean gross error; root mean square error; and FAC2 (i.e., fraction of estimates within a factor of 2).^2,3^

## Results

Table 1 contains estimated correlation coefficients and variance ratios obtained from the validation datasets and used to define our scenarios. It illustrates that in a real-world example, the spatial and temporal variance ratios may differ quite markedly as can the spatial and temporal correlation coefficients. The hybrid1 model provided out-of-plausible range predictions for one roadside/kerbside O_3_ monitoring site, resulting in a large spatial variance ratio and a small negative spatial correlation coefficient (Table 1).

**Table 1.**
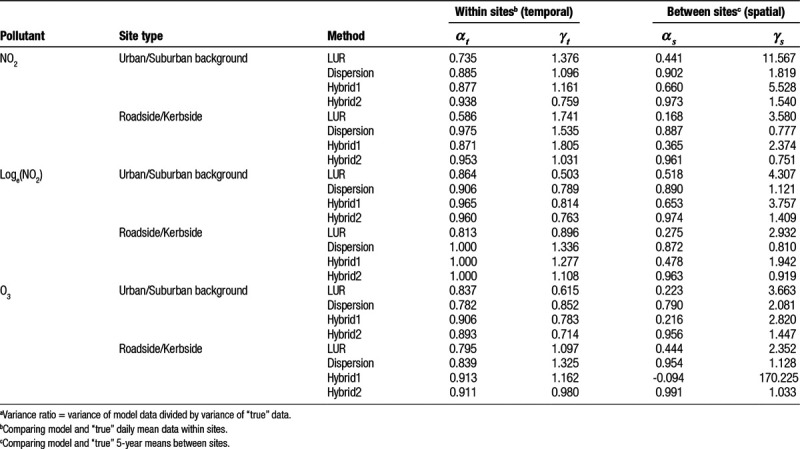
Estimates of correlation coefficients 
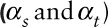
 and variance ratios^a^ 
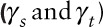
 comparing model and “true” data within sites and between sites, respectively.

### Simulation results

Simulation findings for all-cause mortality are summarized in Tables 2–4. For all pollutant-site-type scenarios, the LUR exposure estimates produced a sizeable downward bias in the estimated health effect of long-term exposure ranging from −91% for roadside/kerbside NO_2_ to −68% for roadside/kerbside O_3_. For short-term exposure, bias also tended to be negative though not as large (i.e., −56% to −23%), although for urban/suburban O_3_, bias was small and positive (4%). When dispersion exposure estimates were used, negative biases were generally smaller, substantially in some cases, and the previously positive bias for short-term exposure to urban/suburban O_3_ became negative (−18%). Including dispersion outputs as an additional covariate in the LUR model produced out-of-plausible exposure range predictions for one roadside/kerbside O_3_ monitoring site and only marginal improvements in health effect estimation for other pollutant site-type combinations. However, combining both LUR and dispersion predictions in a generalized additive model tended to minimize bias in health effect estimates, which ranged from −28% to 11% for long-term exposure and −17% to 11% for short-term exposure.

**Table 2.**
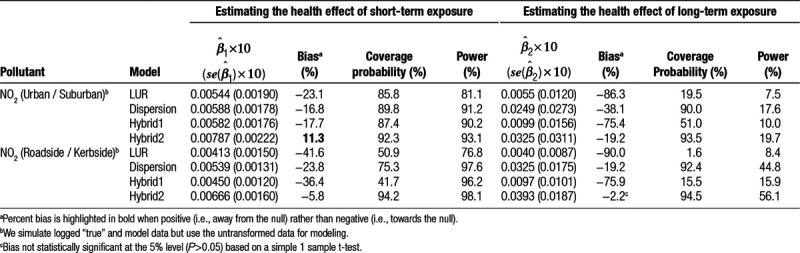
All-cause mortality and NO_2_ (measurement error: additive): 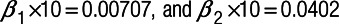.

**Table 3.**
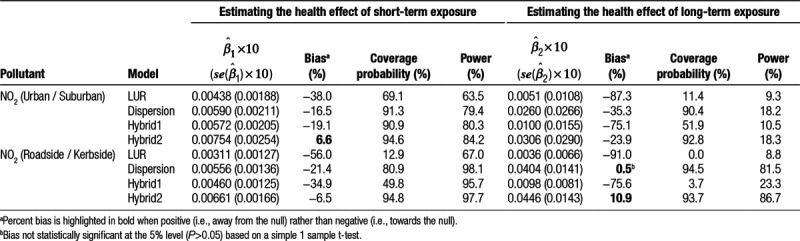
All-cause mortality and NO_2_ (measurement error: proportional): 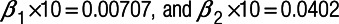.

**Table 4.**
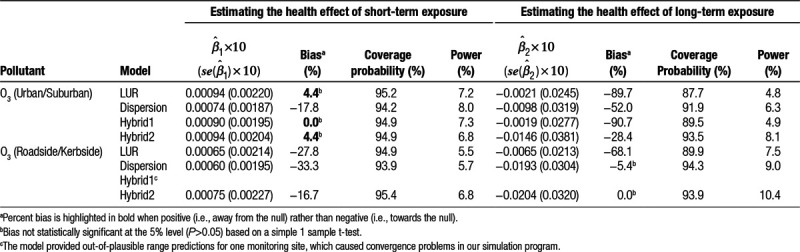
All-cause mortality and O_3_ (measurement error: additive): 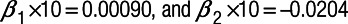.

The hybrid2 model also appeared optimal for coverage probability and statistical power, with values of the former ranging from 92.3% to 95.4% and values of the latter for short-term exposure to NO_2_ ranging from 84.2% to 98.1%. For long-term exposure, due to smaller sample size, and for short-term exposure to O_3_, due to a very small CRF, statistical power was much lower but was nevertheless, with one exception, (short-term exposure to urban/suburban O_3_) higher for the hybrid2 model than for the other modeling approaches. For the dispersion model, the lowest (worst) coverage probability was 75.3% observed for short-term exposure to roadside/kerbside NO_2_ (proportional error), although the corresponding figure for long-term exposure was 92.4%. For LUR, coverage probabilities for O_3_ scenarios were greater than 87%. However, this was not the case for NO_2_, especially for long-term exposure at roadside/kerbside sites, where a coverage probability of 0% was obtained.

Results from our simulations based on hospital admissions for cardiovascular disease can be found in eTables 2–4; http://links.lww.com/EE/A86; and exhibit similar patterns to mortality.

#### Standard performance metrics

Validation statistics of mean bias, normalized mean bias, normalized gross mean error, root mean square error, and FAC2 (Table 5) also favored the hybrid2 model. Nevertheless, it is noteworthy that the LUR model produced the lowest mean bias for roadside/kerbside NO_2_ (i.e., the smallest absolute difference between modeled and measured daily mean NO_2_ concentrations averaged across roadside/kerbside sites).

**Table 5.**
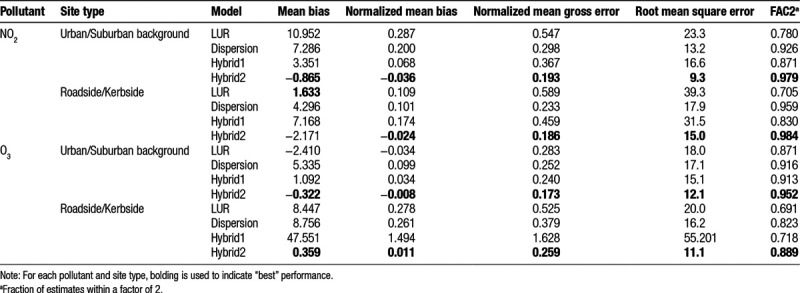
Standard validation statistics calculated for each monitoring site and then averaged over sites.

## Discussion

### Summary of findings and context

We find that with either additive or multiplicative error, the bias induced in health studies is negative (i.e., towards the null) and generally substantially negative. From our simulation results and standard performance metrics, the hybrid2 model combining LUR and dispersion predictions was the preferred choice for use in a multilevel analyses of air pollution and health within the London area, in terms of minimizing the downward bias.

Standard measurement error theory considers two error types, that is, classical and Berkson.^34^ Additive classical error is evidenced by a high variance ratio (model versus “true”) and generally leads to downward bias in health effect estimates, underestimation of standard errors and reduced coverage of 95% confidence intervals, whereas pure additive Berkson error is evidenced by a low variance ratio (model versus “true”) and results in inflated standard errors and reduced statistical power.^34,35^ However, measurement error introduced into modeled air pollution data may be more complex. This has led Szpiro et al,^1^ in the context of LUR modeling, to describe classical-like error (i.e., behaving like classical error) introduced by parameter estimation and Berkson-like error introduced by oversmoothing. Given that total measurement error depends not only on the variances of both modeled and “true” data but also on their covariance, it is important to consider not only the variance ratio (model versus “true”) but also the correlation coefficient (model versus “true”) when assessing the impact of both classical/classical-like and Berkson/Berkson-like error in an epidemiological analysis.^5^ Here and in line with the findings of our previous simulation work,^5^ we observed some small bias away from the null when a high correlation was paired with a low variance ratio and substantial bias towards the null when a high variance ratio was paired with a low correlation coefficient (Tables 1–4).

Based on our simulations, the LUR model predictions performed well for short-term exposure to urban/suburban O_3_, producing only a small positive bias in the health effect estimate, although for long-term exposure bias was large and negative. For scenarios involving NO_2_, the dispersion model rather than the LUR model consistently produced lower bias, higher coverage probability, and higher statistical power.

For NO_2_, which is often found to have a positive skew distribution, we explored the effects of both additive and proportional measurement error, but contrary to some other simulation studies,^6,7^ observed few differences in our results (see Tables 2 and 3). However, when we plotted histograms of site-mean corrected NO_2_ measurements by site type, we observed little positive skew, which may explain these findings.

Some writers have argued that substantial upward bias can result from measurement error in air pollution studies. For example, Crump^36^ conducted simulation studies in linear regression and reported upward bias with proportional measurement error, whereas we generally observed downward bias in our simulations with proportional error. We think this likely reflects his focus on a restricted set of dose-response relationships (Y~bX^n^), whereas our analysis examines the more usual case of a log-linear relationship.

Standard metrics of exposure error, such as mean bias, which address the issue of how closely the model predicts true exposure on a daily basis, provide limited insight into the magnitude of biases introduced into a complex epidemiological analysis and may, in some instances, be misleading. For example, in Table 5, for roadside/kerbside NO_2_, the LUR model produced the smallest mean bias, and yet, our simulations indicate that its use in a multilevel analysis of air pollution and health, leads to substantial underestimation of health effect estimates for both short-term and long-term exposure, poor coverage probabilities, and low statistical power. Nevertheless, when various standard metrics were viewed as a whole, they supported our overall conclusion.

### Possible explanations

Given our validation data compares modeled output to monitoring data and is, therefore, focused on a point (i.e., the coordinates of the monitoring station), we might expect the LUR model to have an advantage. However, the LUR is trained at monitoring sites whose distribution is not random, and this may provide a disadvantage for predictions at other locations, including held out monitoring stations. Further, the dispersion model predicts to a high level of spatial resolution (i.e., 20 m) and then estimates pollutant exposure at a point using bilinear interpolation. The high spatial resolution of the dispersion model and the use of the 10% leave-out method for the LUR model may explain part of our findings, although the fact that the dispersion model performed better overall especially with respect to the traffic-related pollutant (NO_2_) may suggest that the LUR is simply missing some potentially important covariates or more complex associations between those considered. Nevertheless, as Szpiro et al^1^ found in their simulation study, simply dropping an important variable from a correctly specified LUR may have little impact on health effect bias, as any loss of prediction accuracy may be counter-balanced by a reduction in the amount of classical measurement error introduced through model parameter estimation.

When a spline in the dispersion output was added to the LUR model as a covariate, the overall improvement in performance was marginal. The superiority of hybrid2, therefore, suggests that the performance of both LUR and dispersion outputs may not be uniform across the range of pollutant exposures and that combining them using penalized splines within a GAM facilitates better compensation of one for the deficiencies of the other. Di et al^37^ has recently reported that using penalized splines to ensemble average different predictors for particulate matter of diameter <2.5 μm also reduced error precisely because the relative fit between models changed with concentration.

### Study strengths and limitations

The statistical model used within our simulations enabled us to estimate the within-LSOA effect of short-term exposure and the between-LSOA effect of long-term exposure. Details of the model and a consideration of its strengths and limitations can be found in the original article by Kloog et al.^10^

It is possible that some bias observed in our health effect estimates is an artifact of random error introduced by the simulation procedure itself. However, this bias is likely to be small, as evidenced from our one-sample t-tests for all-cause mortality (Tables 2–4), which were significant for all bias estimates >4.4% away from the null or >5.4% towards the null.

One advantage of our study is that we tried to evaluate and correct for classical measurement error in the day to day monitored data so that the variance ratios and correlation coefficients used in our simulations better-reflected comparisons between modeled and “true” data as opposed to modeled and monitored data.^5,7,8^ Having generated “true” data with given spatio-temporal variation and spatial covariance, we then simulated pseudo-modeled data from the “true” by using these metrics (i.e., the correlation coefficients and variance ratios) to introduce measurement error (see ePage 7; http://links.lww.com/EE/A86 for checks on simulations). This approach did not specifically allow for the fact that measurement error introduced by spatio-temporal modeling maybe both heteroscedastic and spatially correlated.^38^ Nevertheless, some of the variance ratio / correlation coefficient combinations obtained from the validation study naturally introduced a lack of independence between the Berkson component and pseudo-modeled data and / or the classical component and “true” data. One limitation of our approach is that it does not provide insight into the effects of including covariates in the analysis, which, if correlated with the pollutant of interest, may lead to additional bias in health effect estimation. The nature of this bias depends on many factors, including the type of error in the pollution data (i.e., classical, Berkson, additive, proportional), whether the covariates are themselves measured with error, the relationship between the pollutant data and the covariates, and whether their respective measurement errors are correlated.^39^ Thus, although some of these issues have been considered by other simulation studies,^9^ they are very specific to the covariates or combinations of covariates to be included and whether the same covariates have been used in developing the air pollution model e.g. temporal covariates in LUR models.

## Conclusions

Although our study is confined to the London area and four examples of different modeling approaches, it illustrates how the choice of air pollution model or combination thereof can be informed by using simulation as well as more conventional validation metrics.

## Conflict of interest statement

The authors declare that they have no conflicts of interest with regard to the content of this report.

## ACKNOWLEDGMENTS

We are grateful to the UK Met Office for provision of meteorological data, accessed through the Centre for Environmental Data Analysis (CEDA). We are also grateful to the UK Government and Local Authorities providing air pollution measurements used in this study, managed by King’s College London and Ricardo Energy and Environment. B.K.B. analyzed the validation data, conducted the simulations, and took the lead in designing the simulations and drafting the article. E.S. contributed to the simulation design. B.B. constructed the monitoring dataset. S.D.B. and N.K. constructed the dispersion model, and K.D. constructed the LUR and hybrid models. S.D.B., N.K., and K.D. used their respective models to produce pollutant predictions at fixed monitoring sites. K.K., R.W.A., B.B., S.D.B., E.S., J.W.S., and B.K.B. were involved in the study design. All authors contributed to the drafting of the article, read and approved the final version.

## Supplementary Material


