# Supplementary material for: Comparing the performance of air pollution models for nitrogen dioxide and ozone in the context of a multilevel epidemiological analysis
Source: Environ Epidemiol. 2020 May 13;4(3):e093. doi: 10.1097/EE9.0000000000000093 (PMC7319188; doi:10.1097/EE9.0000000000000093)
Supplement: Supplementary file 1 [file ee9-4-e093-s001.docx]

**eAppendix**

Contents:

E-Table 1**:** Baseline disease rates and concentration response functions used in simulations

E-Page 3: Estimating the Pearson correlation coefficients and variance ratios used to define our simulation scenarios.

E-Table 2: Cardiovascular hospital admissions and NO_2_ (measurement error: additive)

E-Table 3: Cardiovascular hospital admissions and NO_2_ (measurement error: proportional)

E-Table 4: Cardiovascular hospital admissions and O_3_ (measurement error: additive)

E-Page 7: Checks on simulations

E-Table 5: Checks on correlations and variance ratios incorporated in pseudo-modelled roadside / kerbside NO_2_ data.

E-Pages 8-10: References

**E-Table 1:** Baseline disease rates (c_3_) and concentration response functions for short-term ($\beta_{1}$) and long-term $(\beta_{2})$ exposure used in our simulations.

| **Disease**  **Outcome** | **Baseline rate per LSOA per day**  $\boldsymbol{exp}\boldsymbol{(}\boldsymbol{c}_{\boldsymbol{3}}\boldsymbol{)}$ | **Pollutant** | **Concentration response function per 1 µg/m^3^** | |
| --- | --- | --- | --- | --- |
|  |  |  | **Short-term exposure**  $\boldsymbol{(}\boldsymbol{\beta}_{\boldsymbol{1}}\boldsymbol{)}$ | **Long-term exposure**  $\boldsymbol{(}\boldsymbol{\beta}_{\boldsymbol{2}}\boldsymbol{)}$ |
| All-cause Mortality | 0.0264^a^ | NO_2_ | 0.000707^b^ | 0.00402^c^ |
|  |  | O_3_ | 0.000090^d^ | -0.00204^e^ |
| Cardiovascular hospital admissions | 0.0835^f^ | NO_2_ | 0.000419^b^ | 0.00583^g^ |
|  |  | O_3_ | 0.000539^d^ | -0.00207^hi^ |

^a^ Average death rate per LSOA per day in London in 2011 estimated using data from the Office for National Statistics[1,2]; ^b^ Mills et al, 2015[3]; ^c^ Faustini et al, 2014[4]; ^d^ Katsouyanni et al, 2009[5]; ^e^ Atkinson et al, 2016[6] ; ^f^ Number of hospital admissions per LSOA per day for the financial year 2011-2012 estimated using data from the Office for National Statistics,[1] and NHS Digital[7]; ^g^ Katsoulis et al, 2014[8]; ^h^ Halonen et al 2016 [9]; ^i^ Based on the population-weighted average of two age-specific concentration response function using age-specific population data for London 2011 from the Office for National Statistics [10].

Estimating the Pearson correlation coefficients and variance ratios used to define our simulation scenario.

For each pollutant, site-type and pollution model, the validation data provided us with daily mean monitor measurements ($x_{i,t})$ linked to their corresponding model predictions ($w_{i,t})$. We estimated the spatial variance ${(\sigma}_{s}^{2})$ and temporal variance ($\sigma_{t}^{2})$of “true” data (i.e. excluding instrument error) based on an analysis of the monitor measurements, as described in detail in the supplementary material of our previous paper.[11] We then estimated$\alpha_{s}$,$\alpha_{t},\gamma_{s}{\mathrm{and} \gamma}_{t}$ as follows:

$$\alpha_{s}=\frac{(cov(\bar{x}_{i},\bar{w}_{i})}{\sqrt{\sigma_{s}^{2}} \times\sqrt{var(\bar{w}_{i})}}$$

$$\alpha_{t}=\frac{cov(x_{t},w_{t})}{\sqrt{\sigma_{t}^{2}}\times\sqrt{var\left( w_{t} \right)}}$$

$$\gamma_{s}=\frac{var(\bar{w}_{i})}{\sigma_{s}^{2}}$$

$$\gamma_{t}=\frac{var\left( w_{t} \right)}{\sigma_{t}^{2}}$$

Where $var\left( w_{t} \right)$ represents the average within-site variance of the daily modelled pollutant data and $cov\left( x_{t},w_{t} \right)$ the average within-site covariance between daily modelled and measured pollutant data.

**E-Table 2** Cardiovascular hospital admissions and NO_2_ (measurement error: additive): $\beta_{1}\times10=0.00419, \mathrm{and}\beta_{2}\times10=0.0583$

| **Pollutant** | **Model** | **Estimating the health effect of short-term exposure** | | | | **Estimating the health effect of long-term exposure** | | | |
| --- | --- | --- | --- | --- | --- | --- | --- | --- | --- |
|  |  | $\hat{\boldsymbol{\beta}_{\boldsymbol{1}}}\boldsymbol{\times10}$  $\boldsymbol{(se}\mathbf{(}\hat{\boldsymbol{\beta}_{\boldsymbol{1}}}\boldsymbol{) \times10)}$ | **Bias ^a^**  **(%)** | **Coverage probability**  **(%)** | **Power**  **(%)** | $\hat{\boldsymbol{\beta}_{\boldsymbol{2}}}\boldsymbol{\times10}$  $\boldsymbol{(se}\mathbf{(}\hat{\boldsymbol{\beta}_{\boldsymbol{2}}}\boldsymbol{) \times10)}$ | **Bias ^a^**  **(%)** | **Coverage Probability**  **(%)** | **Power**  **(%)** |
| NO_2_  (Urban / Suburban) | Land Use Regression  (LUR) | 0.00267  (0.00102) | -36.3 | 68.8 | 73.6 | 0.0072  (0.0106) | -87.7 | 0.7 | 11.0 |
|  | Dispersion | 0.00357  (0.00115) | -14.8 | 90.2 | 86.9 | 0.0400  (0.0254) | -31.4 | 87.7 | 36.2 |
|  | Hybrid1 | 0.00346  (0.00112) | -17.4 | 88.8 | 86.1 | 0.0167  (0.0153) | -71.4 | 23.7 | 20.4 |
|  | Hybrid2 | 0.00452  (0.00138) | **7.9** | 93.9 | 89.1 | 0.0472  (0.0279) | -19.0 | 92.5 | 40.5 |
| NO_2_  (Roadside / Kerbside) | Land Use Regression  (LUR) | 0.00188  (0.00068) | -55.1 | 8.8 | 78.5 | 0.0051  (0.0066) | -91.3 | 0.0 | 12.4 |
|  | Dispersion | 0.00333  (0.00073) | -20.5 | 78.3 | 99.7 | 0.0584  (0.0137) | **0.2^b^** | 94.4 | 99.1 |
|  | Hybrid1 | 0.00274  (0.00067) | -34.6 | 40.9 | 98.2 | 0.0139  (0.0080) | -76.2 | 0.0 | 41.0 |
|  | Hybrid2 | 0.00397  (0.00089) | -5.3 | 94.5 | 99.6 | 0.0641  (0.0140) | **9.9** | 92.1 | 99.7 |

^a^ Percent bias is highlighted in bold when positive (i.e. away from the null) rather than negative (i.e. towards the null); ^b^ Bias not statistically significant at the 5% level (p>0.05) based on a simple one sample t-test.

**E-Table 3** Cardiovascular hospital admissions and NO_2_ (measurement error: proportional): $\beta_{1}\times10=0.00419, \mathrm{and}\beta_{2}\times10=0.0583$

| Pollutant | Model | Estimating the health effect of short-term exposure | | | | Estimating the health effect of long-term exposure | | | |
| --- | --- | --- | --- | --- | --- | --- | --- | --- | --- |
|  |  | $\hat{\beta_{1}} \times10$  $(se(\hat{\beta_{1}}) \times10)$ | Bias ^a^  (%) | Coverage probability  (%) | Power  (%) | $\hat{\beta_{2}}\times10$  $(se(\hat{\beta_{2}}) \times10)$ | Bias ^a^  (%) | Coverage Probability  (%) | Power  (%) |
| NO_2_  (Urban / Suburban)^c^ | Land Use Regression  (LUR) | 0.00320  (0.00103) | -23.6 | 82.7 | 86.5 | 0.0079  (0.0117) | -86.4 | 1.2 | 10.1 |
|  | Dispersion | 0.00349  (0.00097) | -16.7 | 88.4 | 94.4 | 0.0368  (0.0259) | -36.9 | 86.3 | 32.1 |
|  | Hybrid1 | 0.00344  (0.00096) | -17.9 | 87.0 | 95.0 | 0.0140  (0.0152) | -76.0 | 17.4 | 16.1 |
|  | Hybrid2 | 0.00464  (0.00121) | **10.7** | 92.7 | 96.7 | 0.0472  (0.0297) | -19.0 | 93.4 | 36.1 |
| NO_2_  (Roadside / Kerbside)^c^ | Land Use Regression  (LUR) | 0.00251  (0.00083) | -40.1 | 44.1 | 87.4 | 0.0066  (0.0085) | -88.7 | 0.0 | 13.2 |
|  | Dispersion | 0.00323  (0.00070) | -22.9 | 71.8 | 99.7 | 0.0480  (0.0168) | -17.7 | 88.6 | 79.1 |
|  | Hybrid1 | 0.00271  (0.00064) | -35.3 | 34.2 | 99.1 | 0.0146  (0.0100) | -75.0 | 0.9 | 30.8 |
|  | Hybrid2 | 0.00399  (0.00085) | -4.8 | 95.3 | 99.6 | 0.0575  (0.0181) | -1.4^b^ | 93.7 | 87.0 |

^a^ Percent bias is highlighted in bold when positive (i.e. away from the null) rather than negative (i.e. towards the null); ^b^ Bias not statistically significant at the 5% level (p>0.05) based on a simple 1 sample t-test; ^c^ We simulate logged true and model data but use the untransformed data for modelling.

**E-Table 4** Cardiovascular hospital admissions and O_3_ (measurement error: additive): $\beta_{1}\times10=0.00539, \mathrm{and}\beta_{2}\times10=-0.0207$

| Pollutant | Model | Estimating the health effect of short-term exposure | | | | Estimating the health effect of long-term exposure | | | |
| --- | --- | --- | --- | --- | --- | --- | --- | --- | --- |
|  |  | $\hat{\beta_{1}} \times10$  $(se(\hat{\beta_{1}}) \times10)$ | Bias ^a^  (%) | Coverage probability  (%) | Power  (%) | $\hat{\beta_{2}}\times10$  $(se(\hat{\beta_{2}}) \times10)$ | Bias ^a^  (%) | Coverage Probability  (%) | Power  (%) |
| O_3_  (Urban / Suburban) | Land Use Regression  (LUR) | 0.00577  (0.00124) | **7.1** | 93.9 | 99.6 | -0.0004  (0.0234) | -98.1 | 83.0 | 7.1 |
|  | Dispersion | 0.00457  (0.00105) | -15.2 | 89.0 | 98.8 | -0.0106  (0.0294) | -48.8 | 88.5 | 10.6 |
|  | Hybrid1 | 0.00555  (0.00110) | **3.0** | 93.6 | 99.9 | -0.0025  (0.0261) | -87.9 | 88.1 | 7.7 |
|  | Hybrid2 | 0.00573  (0.00115) | **6.3** | 95.1 | 99.9 | -0.0156  (0.0347) | -24.6 | 90.2 | 12.0 |
| O_3_  (Roadside / Kerbside) | Land Use Regression  (LUR) | 0.00404  (0.00121) | -25.0 | 79.7 | 92.1 | -0.0057  (0.0207) | -72.5 | 87.5 | 5.0 |
|  | Dispersion | 0.00392  (0.00110) | -27.3 | 72.5 | 94.9 | -0.0191  (0.0291) | -7.7^b^ | 93.0 | 11.6 |
|  | Hybrid1^c^ |  |  |  |  |  |  |  |  |
|  | Hybrid2 | 0.00491  (0.00128) | -8.9 | 93.4 | 96.9 | -0.0200  (0.0309) | -3.4^b^ | 93.3 | 11.0 |

^a^ Percent bias is highlighted in bold when positive (i.e. away from the null) rather than negative (i.e. towards the null); ^b^ Bias not statistically significant at the 5% level (p>0.05) based on a simple 1 sample t-test; ^c^The model provided particularly poor predictions for one monitoring site, which caused convergence problems in our simulation program

**Checks on simulations**

The ability of our simulation programs to produce “true” and pseudo-modelled data with given correlations and variance ratios both spatially and temporally was assessed by including checks within our simulation program for roadside / kerbside NO_2_ (additive error).

Overall the correlations and variance ratios, when averaged across simulations for roadside / kerbside NO_2_ (additive error) were consistent with their target values (E-Table 5). There was a slight positive bias in the spatial variance ratio but this was negligible (<0.4%).

**E-Table 5:** Checks on correlations and variance ratios incorporated in pseudo-modelled roadside / kerbside NO_2_ data.

| Temporal / Spatial | True / model | Variance | | Variance Ratio | | Correlation Coefficient | |
| --- | --- | --- | --- | --- | --- | --- | --- |
|  |  | Simulated^a^ | Specified | Simulated^b^ | Specified | Simulated^c^ | Specified |
| Temporal | True | 359.0 | 359.1262 | 1 | 1 | 1 | 1 |
|  | LUR | 625.2 |  | 1.743 | 1.741 | 0.586 | 0.586 |
|  | Dispersion | 551.1 |  | 1.535 | 1.535 | 0.975 | 0.975 |
|  | Hybrid1 | 648.1 |  | 1.806 | 1.805 | 0.871 | 0.871 |
|  | Hybrid2 | 370.2 |  | 1.031 | 1.031 | 0.953 | 0.953 |
| Spatial | True | 653.4 | 654.5549 | 1 | 1 | 1 | 1 |
|  | LUR | 2337 |  | 3.593 | 3.580 | 0.168 | 0.168 |
|  | Dispersion | 506.9 |  | 0.777 | 0.777 | 0.887 | 0.887 |
|  | Hybrid1 | 1551 |  | 2.383 | 2.374 | 0.364 | 0.365 |
|  | Hybrid2 | 490.9 |  | 0.752 | 0.751 | 0.961 | 0.961 |

^a^ Average within-simulation variance. ^b^ Average within-simulation variance ratio. ^c^ Average within-simulation correlation.

LUR: Land Use Regression. Hybrid 1: LUR with dispersion output spline as a covariate. Hybrid 2: generalised additive model (GAM) combining splines in LUR and dispersion outputs.

**References**

[1] Office for National Statistics. 2011 Census: Usual residents by resident type, and population density, number of households with at least one usual resident and average household size, Output Areas (OAs) in London. <https://www.ons.gov.uk/peoplepopulationandcommunity/populationandmigration/populationestimates/datasets/2011censuspopulationandhouseholdestimatesforwardsandoutputareasinenglandandwales>. Accessed August 22, 2017. The data are © Crown Copyright 2012, licenced under the Open Government Licence v3.0. [https://www.nationalarchives.gov.uk/doc/open-government-licence/version/3/](http://www.nationalarchives.gov.uk/doc/open-government-licence/version/3/).

[2] Office for National Statistics‚ National Records of Scotland‚ Northern Ireland Statistics and Research Agency. Mortality Statistics: Deaths registered by area of usual residence, 2011 registrations. <https://www.ons.gov.uk/peoplepopulationandcommunity/birthsdeathsandmarriages/deaths/datasets/deathsregisteredbyareaofusualresidenceenglandandwales>. Accessed August 21, 2017. The data are © Crown Copyright 2013, licenced under the Open Government Licence (OGL) v3.0. <https://www.nationalarchives.gov.uk/doc/open-government-licence/version/3/>.

[3] Mills IC, Atkinson RW, Kang S, Walton H, Anderson HR. Quantitative systematic review of the associations between short-term exposure to nitrogen dioxide and mortality and hospital admissions. *BMJ Open*. 2015;5:e006946. (Tables S3 and Table 2) doi:10.1136/bmjopen-2014-006946.

[4] Faustini A, Rapp R, Forastiere F. Nitrogen dioxide and mortality: review and meta-analysis of long-term studies. *Eur Respir*. *J* 2014;44:744-753.

[5] Katsouyanni K, Samet JM, Anerson HR et al. *Air pollution and Health: A European and North American Approach (APHENA). HEI Research Report 142*. Boston, MA: Health Effects Institute; 2009. (Pages 31 and 43, Tables 14 and 33: Lag 1, partial autocorrelation function, natural spline model).

[6] Atkinson RW, Butland BK, Dimitroulopoulou C et al.. Long-term exposure to ambient ozone and mortality: a quantitative systematic review and meta-analysis of evidence from cohort studies. *BMJ Open*. 2016;6:e009493. (Table 2) doi:10.1136/bmjopen-2015-009493.

[7] NHS Digital. Hospital Episode Statistics Admitted Patient Care - England, 2011-12: Provider-level analysis. Table A: “Headline figures for England, SHA and individual provider (2011-12)” and Table E: “Finished admission episodes by primary diagnosis chapter for England, SHA and individual provider (2011-12)”. <https://digital.nhs.uk/data-and-information/publications/statistical/hospital-admitted-patient-care-activity/hospital-episode-statistics-admitted-patient-care-england-2011-12>. Source: Hospital Episode Statistics, HES. The Health and Social Care Information Centre. Information from NHS Digital, licenced under the current version of the Open Government Licence. Accessed August 28, 2018.

[8] Katsoulis M, Dimakopoulou K, Pedeli X et al. Long-term exposure to traffic-related air pollution and cardiovascular health in a Greek cohort study. *Sci Total Environ*. 2014;490;934-940. (Table 2)

[9] Halonen JI, Blangiardo M, Toledano MB et al. Long-term exposure to traffic pollution and hospital admissions in London. *Environ Pollution*. 2016;208:48-57.

[10] Office for National Statistics. 2011 Census: Population Estimates by five-year age bands, and Household Estimates, for local Authorities in the United Kingdom. Table P01UK. <https://www.ons.gov.uk/peoplepopulationandcommunity/populationandmigration/populationestimates/datasets/2011censuspopulationestimatesbyfiveyearagebandsandhouseholdestimatesforlocalauthoritiesintheunitedkingdom> . Source: Office for National Statistics, Northern Ireland Statistics and Research Agency, National Record of Scotland. Accessed August 28, 2018. The data are © Crown Copyright 2012, licenced under the Open Government Licence (OGL) v3.0. <https://www.nationalarchives.gov.uk/doc/open-government-licence/version/3/>.

[11] Butland BK, Samoli E, Atkinson RW, Barratt B, Katsouyanni K. Measurement error in a multi-level analysis of air pollution and health: a simulation study. *Environ Health*. 2019;18:13. doi:10.1186/s12940-018-0432-8.
